# Accelerometry-Based Distance Estimation for Ambulatory Human Motion Analysis

**DOI:** 10.3390/s18124441

**Published:** 2018-12-15

**Authors:** Juan Carlos Alvarez, Diego Álvarez, Antonio M. López

**Affiliations:** Multisensor Systems and Robotics Group (SiMuR), Department of Electrical, Electronic, Computer and Systems Engineering, University of Oviedo, C/ Pedro Puig Adam, 33203 Gijón, Spain; dalvarez@uniovi.es (D.Á.); amlopez@uniovi.es (A.M.L.)

**Keywords:** microelectromechanical inertial sensors, measurement uncertainty, accelerometers, distance estimation

## Abstract

In human motion science, accelerometers are used as linear distance sensors by attaching them to moving body parts, with their measurement axes its measurement axis aligned in the direction of motion. When double integrating the raw sensor data, multiple error sources are also integrated integrated as well, producing inaccuracies in the final position estimation which increases fast with the integration time. In this paper, we make a systematic and experimental comparison of different methods for position estimation, with different sensors and in different motion conditions. The objective is to correlate practical factors that appear in real applications, such as motion mean velocity, path length, calibration method, or accelerometer noise level, with the quality of the estimation. The results confirm that it is possible to use accelerometers to estimate short linear displacements of the body with a typical error of around 4.5% in the general conditions tested in this study. However, they also show that the motion kinematic conditions can be a key factor in the performance of this estimation, as the dynamic response of the accelerometer can affect the final results. The study lays out the basis for a better design of distance estimations, which are useful in a wide range of ambulatory human motion monitoring applications.

## 1. Introduction

Since seminal work by from Morris [1], MEMS accelerometers and gyroscopes have been increasingly used for the real-time measurement of body motion spatio-temporal parameters, due to their low consumption and cost, and their easy connectivity. Specifically, they are the base component for wearable devices that measure linear and/or angular displacements in the human body, e.g., the shank rotation, the stride length, and the pelvis displacement. The stride length is needed, for example, in pedestrian navigation systems [2] or for clinical gait analysis [3]. The pelvis displacement is useful in rehabilitation or prosthetics, as an indicator of the metabolic cost in walking [4], to discriminate left and right steps [5], or to estimate the step length [6,7,8].

Although particularly convenient for real-time applications, these MEMS-based estimations suffer the problem of an unbounded error growth with time [9]. For angular displacements, the problem is usually addressed by the sensor integration of gyroscopes, accelerometers, and magnetic field sensors, by means of Kalman-filtering-like algorithms, forming inertial measurement units (IMU) [10].

However, for linear displacements, there is no unique accepted solution. The basic principle of accelerometers as linear position sensors is straightforward: the measured acceleration is converted to a linear position by doubly integrating the accelerometer data, that is, by adding up a noisy signal coming from a sensor supposedly aligned in a measurement axis. However, error grows too fast with time, making it mandatory to provide some kind of error compensation. Otherwise, the results can be unacceptable for most applications, even if integration is made in short time slots.

The main error source comes from sensor bias and the duration of the integration time. Bias is the difference between the sensor output and the true value and it has multiple origins, both deterministic and stochastic. Deterministic bias sources are those that could be predicted, and possibly corrected by a per device calibration. On the contrary, stochastic bias sources can be considered random and need to be compensated. They are probabilistically modeled, in the form of power spectral density or Allan variance, and they have a variety of origins: velocity random walk (additive white noise), bias instability (flicker noise), quantization noise, sinusoidal errors, rate random walk, and rate ramp [11]. A possible general model of the accelerometry measurement process could be

y(t)=S·a(t)+ϵ(T)+N(a,T,t)+e(t)

y(t) being the sensor output, a(t) the real acceleration to be measured, *S* the scale factor, ϵ(T) a temperature-dependent bias, N(a,T,t) a time-dependent non-linear bias function, and e(t) the bias error caused by stochastic sources [12]. If a certain bias in acceleration is integrated, it will produce an error in distance estimation which grows quadratically with the integration time [13]. Then, integration time is a key parameter to consider when studying distance estimation errors.

In spite of the widespread use of this measurement technique in real-time human-motion-related applications, there is a lack of agreement about how to define and compensate its errors. The error-correction methods proposed in the literature are heuristic in nature and do not provide a general design criteria. It is not clear how different error sources behave with integration time [9] or how to evaluate the relative weight of each source in the final results.

In this paper, we aim to shed try to put some light on this problem by making a systematic comparison of different distance estimation methods with different sensors, to check if they are compatible with the demands imposed by ambulatory human motion monitoring applications: accuracy in real-time and robustness to various experimental kinematic conditions.

The presented results confirm that it is possible to use accelerometers to estimate short linear displacements of the body with a 4.5% error in typical conditions. However, it is also shown that the motion conditions, such as mean velocity and acceleration profiles, can be a key factor in the performance of this estimation, as the dynamic response of the accelerometer can affect the results. The study sets out the basis for a systematic design of distance estimations, and it provides a tool to better interpret their results depending on the experimental conditions.

Section 2 will develop the distance estimation problem and its experimental conditions in the context of human motion science. Section 3 will detail the estimation methods selected and the design and conditions of the experiments. The results will be explained in Section 4 and discussed in Section 5. The main conclusions will be synthesized in Section 6.

## 2. Real-Time Distance Estimation for Cyclic Motion

In human movement science (HMS), most distance estimations of interest are not the result of a free linear motion, but rather they are related to cyclical or periodical motions, such as human walking. For example, the body center of mass moves up (vCOM) and sideways (mlCOM) to its maximum twice each stride in normal gait or the distance traveled by one foot between two consecutive heel strike events, which defines the stride length (SL).

**Figure 1:**
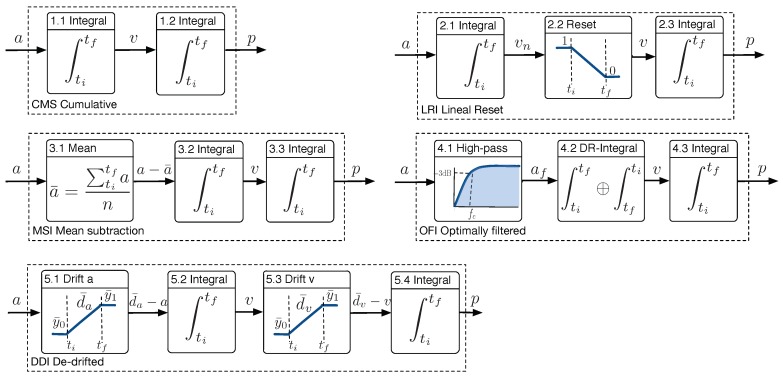
Estimation methods compared in this work.

Several distance estimation methods have been proposed in the human movement science literature. In spite of its known limitations, the simple direct cumulative double integration (CMS) method has been applied to estimate the stride length in devices for the ambulatory analysis of gait [14]. We will test this method as a worst-case scenario for distance estimation. Other methods, see Figure 1, try to mitigate the growth of error caused by the direct signal integration, because they are noticeable even within the short time integration periods of interest (0.4–1.6 s).

In Ref. [15], the direct method is slightly modified for both horizontal and vertical motion of the foot, to estimate the step length. Previous to the second integral, a lineal resetting mechanism is applied to the velocity by weighting linearly between 1 and 0 during the integration time (LRI method). This is to ensure that v(T)=0, a biomechanics property that both foot velocities were supposed to have. The authors claim that the aim of the method is to remove measurement error due to noise and drift.

An integration with mean subtraction (MSI method) is proposed in Ref. [8] to estimate zCOM displacements from COM accelerations, and in Ref. [16] for foot displacements, both in the context of gait analysis. The idea was to remove the non-zero medium value ${\bar{a}}_{s}\neq 0$ caused by sensor artifacts and uncertainties, prior to the first velocity integration. The mean value of the acceleration between two consecutive integration events (ipso-lateral heel strikes) ${\bar{a}}_{s}$ was subtracted, and a first integration was made to estimate the instantaneous velocity. Notice that forcing the acceleration to have a zero mean value is equivalent to assuming that the velocity is the same at the beginning and the end of the segment, for example, if the motion comes to rest at the end of the period,

(1) $$v\left(t\right)=v\left(0\right)+\int_{0}^{T}\left(a_{s}\left(\tau \right)-{\bar{a}}_{s}\right)d\tau$$

${\bar{a}}_{s}$ being the average acceleration in the segment period, and ${\bar{a}}_{s}=\frac{1}{T}\int_{0}^{T}a_{s}\left(\tau \right)d\tau$. In Ref. [8], the mean subtraction was applied twice in the particular case where the final position is the same at the beginning and end of the integration period, but this case will not be addressed here.

In Ref. [17], a technique is presented to estimate the stride length by integration of the body antero-posterior (forward) acceleration, measured at the pelvis. It is denominated as the optimally filtered integration (OFI method). To reduce the low frequency acceleration noise, at each gait cycle, the acceleration signal was high-pass-filtered, with a 2nd order Butterworth filter with an $f_{c}$ Hz cut frequency, calculated from data of a given gait cycle with known equal initial and final velocities. After filtering, a first integration produced instantaneous velocity. Taken from Ref. [18], the numerical method chosen to integrate the data was the Cavalieri–Simpson. If the so-estimated final velocity is close to the initial velocity, they are supposed as equal, and a weighted direct and reverse integration is applied, aimed to reduce the time drift in velocity. Notice that this method, contrary to the LRI and MSI methods, did not need a general assumption about the velocity at the end of the integration time, because the authors were dealing with a biomechanics problem (apCOM) with different characteristics.

A different method is proposed in Ref. [19], named de-drifted integration (DDI method). It encompasses the subtraction of a weighted mean function of data samples, previous to each of both integrations. In this case, it was applied to instep foot accelerations during a stride time cycle, with no previous filters, to estimate the stride length. The acceleration drift function was computed by averaging a few initial and final accelerometry samples and by removing a lineal interpolation between both values from the original signal. The size of both sets of samples was empirically selected. The velocity drift function was based on the assumption of zero velocity at the beginning and end of the step, and a lineal function from zero to the mean of the last velocity samples was subtracted from to achieve it.

Other methods have been proposed in human motion analysis for distance estimation that will not be included in this study. In Ref. [3], zCOM and mlCOM displacements are estimated in two steps: (1) filtering the sensor data before the double integration with a low-pass 4th order zero-lag Butterworth filter, at a 20 Hz cut frequency, to remove acceleration noise and (2) filtering the distance estimation after the double integration with a high-pass 4th order zero-lag Butterworth filter, at a 0.1 Hz cut frequency, to remove its long-term time drift. This drift filtering has to be made in longer time intervals (30 s in Ref. [3] or 12–18 s in Ref. [7]). These are useful solutions for off-line data analysis, but not feasible for real-time step-to-step applications.

## 3. Materials and Methods

To evaluate and compare the estimation methods presented, a set of experiments was designed. Inertial sensors were attached to a robot arm programmed with a set of motions. The robot position was stored synchronized with the sensor readings. The data were processed with all methods, and their results were evaluated against the actual robot displacement, which provided the ground truth.

The robot was a six-degree-of-freedom industrial manipulator IRB-120 from ABB, reaching 580 mm with a maximum payload of 3 kg and a position repeatability of 0.01 mm. It was fixed to a workbench and previously calibrated. This robot provided a worst-case linear trajectory repeatability of 0.07 mm (a nominal maximum load of 3 kg and a linear motion with six axes at 1600 mm/s [20]).

To calculate cyclical displacements in real-time for ambulatory applications, they have to be estimated in one stride or step time period. This time span, or integration time, is very short. For a wide population of 95% of healthy men and women, aged from 13 to 80, the step time in walking can be as long as 0.8 s (a cadence of 75 steps/min), and it can decrease to 0.4 s (cadence of 150 steps/min) when the walking velocity increases [21]. This can occur at free-speed walking velocities in ranges approximately from 0.8 to 1.8 m/s.

Likewise, these parameters are subject to certain kinematic restrictions. For example, the stride length (SL) can be measured from the foot [19] or from the antero-posterior displacement of the body center of mass (apCOM) [17]. The average stride length (or two steps) increases with walking speed, varying in the interval of 94–185 cm for the same wide population and the same walking speed ranges mentioned previously [21]. These motions are made with accelerations around 2–5 g [22]. Likewise, trunk vertical excursion (vCOM) is known to increase with walking velocity, roughly from 2.2 to 6 cm at cadences between 66 to 120 steps/s [23] or step times of 0.5–0.9 s. It is also reasonable to hypothesize that vCOM initial and final positions at every stride are the same, which makes its average velocity zero. Inversely, the COM medio-lateral displacement (mlCOM) decreases with walking velocity, in the range from 8 cm at 66 steps/s to 2.4 cm at 120 steps/s [24]. All these variables affecting the estimation of displacements of interest in HMS are summarized in Table 1, assuming a duration of a gait cycle (step) in the range of 0.4–0.8 s.

From these considerations, it is clear that the problem of MEMS-based distance estimations in human motion science is relevant for integration times from 0.4 s (a fast step time) to 1.6 s (a slow stride time). Within this time rank, motions of interest can be divided into two groups: short range motions of a few centimeters (COM-type) occurring at mean velocities between 1 to 20 cm/s, and large range motions (step-like) with mean velocities ranging between 60 and 230 cm/s. These numbers will be used to define eight experiments covering both types of kinematic conditions. experiments in similar kinematic ranges.

The robot was programmed to make eight types of linear vertical motion in the Cartesian space, which tries to resemble the previously discussed whole scenario of distance estimation in typical human motion applications, summarized in Table 2. A short (4 cm) linear trajectory was executed at increasing speeds {1, 2.5, 5, and 10} cm/s. Similarly, a large (40 cm) trajectory was made with speeds of {10, 25, 50, and 100} cm/s. The short trajectory resembles the conditions of COM-like displacements, and the large trajectory of step-like (half the stride) displacements. Each experiment was repeated 50 times to provide statistical significance. The motion parameters were chosen also to provide the same four integration time scenarios in both trajectories, {4, 1.6, 0.8, and 0.4} s, relevant in the context of biomechanics, as discussed in the previous section. The motion was recorded with margins of 0.05 s before and after it was made, to clearly capture the whole motion, so the effective integration time applied was {4.1, 1.7, 0.9, and 0.5} s. In the case of the fastest motion 100 cm/s (Experiment 8), the integration time was incremented further to 0.625 s forced by the physical limitations of the robot. The Experiments 1 and 4 imply an integration time of τ = 4.1 s, which is outside the scope out of the range of interest of this study. They will be used as a reference, to understand how the conclusions obtained for τ under 2 s extrapolate to larger integration times.

**Figure 2:**
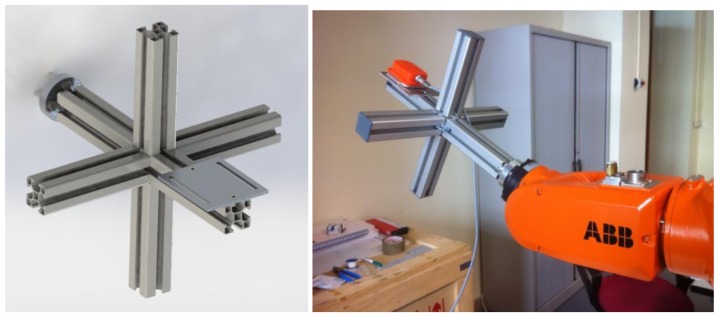
(**Left**) End effector designed to test several sensor units at once. (**Right**) Robot tool motion and sensor attachment.

A custom-designed end effector for the robot was used, which allows one to simultaneously test several accelerometer units, see Figure 2. The units were rigidly attached to the end effector and statically calibrated. Individual calibration was performed with the sensor devices attached and at rest, in the middle point of the linear trajectory.

Three different accelerometers were tested simultaneously, and their nominal characteristics are synthesized in Table 3. Their noise density $\sigma_{c}$ given by the manufacturer was taken as a first indication of the sensor quality. The Accel.XS is part of an MTi IMU device from Xsens^®^ with accelerometers from ADXL206 [25,26]. The Accel.LS is integrated in an IMU unit from Shimmer^®^ with a low-noise analog accelerometer Kionix model KXRB5-2042, which has a better noise performance than the previous model. The Accel.WR is a wide-range accelerometer also included in the previous device, with an LSM303DLHC digital accelerometer from STMicro with the highest nominal noise level of the three [27].

In the three cases, the user can read the raw uncalibrated sensor data with a sampling frequency of $f_{s}$ Hz. However, the setup of the internal signal conditioning, the anti-aliasing filter, and the sampling by the dedicated CPUs are not always available, so it is difficult to know the effective sensor bandwidth $f_{c}$. In the table, a reference value is given, as suggested by the IMU manufacturers and its corresponding RMS noise density level $\sigma_{d}$.

In our experiments, sensor data was recorded at the maximum sampling rate available, $f_{s}=120$ or 512 Hz, depending on the device. Once the sensor was fixed to the end effector, an individual static calibration of its orientation was required. This reorientation was made with the TRIAD method, by using the magnetometer data with the robot at rest [28]. The direction of earth gravitational and magnetic fields (global coordinates) were used to compute two measurements $\left\{{\vec{m}}_{g},{\vec{m}}_{m}\right\}$ in local coordinates, which were converted to a local triad $H_{L}=\left\{{\vec{u}}_{1},{\vec{u}}_{2},{\vec{u}}_{3}\right\}$ by the following process:

(2) $${\vec{u}}_{1}=\frac{{\vec{m}}_{g}}{|{\vec{m}}_{g}|}\;{\vec{u}}_{2}=\frac{{\vec{u}}_{1}\times {\vec{m}}_{m}}{|{\vec{u}}_{1}\times {\vec{m}}_{m}|}\;{\vec{u}}_{3}={\vec{u}}_{1}\times {\vec{u}}_{2}$$

The same process was applied to calculate a global triad $H_{G}$, and both of them allowed us to compute a re-orientation matrix $H_{G}={}^{G}R_{L}H_{L}$. The gravitational field is much more robust and reliable than the magnetic field to be used as a measurement reference, and, for that reason, the robot moved precisely along the vertical axis, and the raw data was reorientated with matrix ${}^{G}R_{L}$. No other data curation is done.

**Figure 3:**
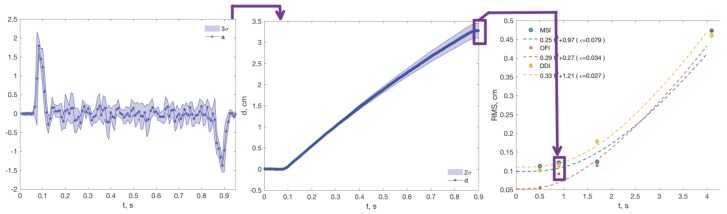
The complete experimental procedure: (**Left**) the accelerometer signals were segmented and analyzed, and the erroneous one was discarded; (**Center**) the estimation methods were applied, and the RMS value was computed at the end of the motion; (**Right**) RMS values were compared for different integration times, sensors, and methods.

The controlled motion of the robot generated an acceleration profile that is similar in the eight experiments described in Table 2. It started with a short acceleration peak, a long period of zero acceleration corresponding to the constant velocity motion, and an inverse symmetrical peak of deceleration to reach zero velocity again, see Figure 3 Left.

Each of the eight experiments in Table 2 was repeated a number of times *M* = 50. The *M* signal segments, sampled with frequency $f_{s}$, were stored and inspected to find artifacts that can occur if samples are lost during a repetition. This was the case for Accel.LS and Accel.WR when sampling at high frequencies (512 Hz). All the signals were inspected to remove those that were non-useful, leading to the discarding of six and seven in 400 repetitions, respectively.

Each of the valid *M* blocks formed a data set, each having *N* samples, accounting for $\tau =N/f_{s}$ s, which corresponds to the integration time for the estimations. Within each block, the data were integrated to obtain a linear position with the five distance estimation methods in Table 4. The error in the last sample (Nth sample) ε(k,N) was calculated for each block k∈M. Then, its root mean square error (RMS) $\bar{\varepsilon }\left(N\right)$ will be used to evaluate the response:

(3) $$\bar{\varepsilon }\left(N\right)=\frac{1}{M}\sqrt{\sum_{k=1}^{M}\varepsilon^{2}\left(k,N\right)}.$$

The complete methodology is represented in Figure 3.

## 4. Results

### 4.1. The Effect of Noise in Distance Estimation

**Figure 4:**
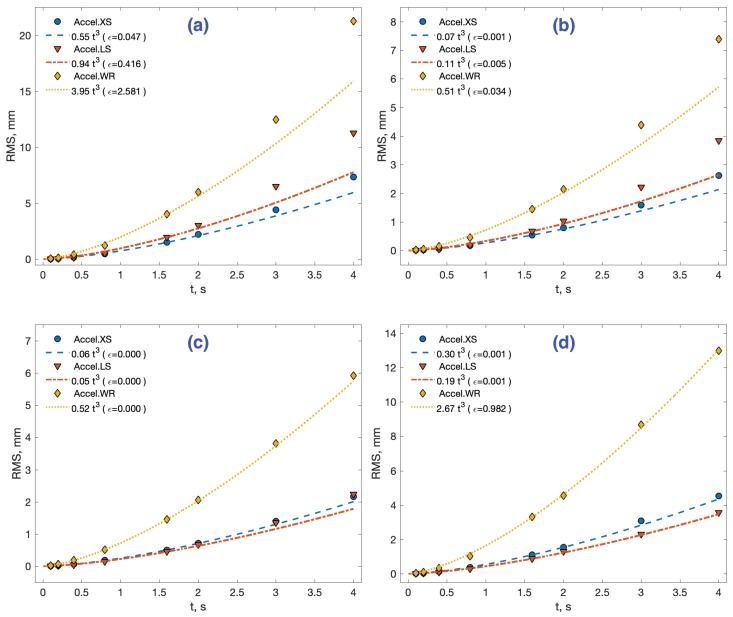
Static RMS distance estimation errors for each sensor (markers) and its cubic polynomial model (lines). (**a**) The CMS method. (**b**) The LRI method. (**c**) The MSI and OFI methods. (**d**) The DDI method. The model fitness is evaluated with statistic ϵ (mm, in parenthesis).

A first experiment was conducted in order to evaluate how different methods cope with sensor noise. To do that, acceleration data were taken from stationary sensors with a sampling frequency $f_{s}$, during 36.95, 37.89, and 50.45 min for each sensor, respectively. Then, data were segmented in groups of fixed time length τ={0.1,0.2,0.4,0.8,1.6,2,3,4} s. This generated 554, 568, and 756 data groups for the 4 s integration time, respectively. The same number of independent groups were used for the rest of the integration times. The estimation methods of interest in Table 4 were applied, and the RMS value of the maximum distance observed in the period, ($\bar{\varepsilon }\left(N\right)$) in Equation (3), was computed. The results are stored in Appendix A, Table A1. Figure 4 represents these results for each method, with different markers for the three sensors. The lines represent a polynomial interpolation made with four central data corresponding to integration times of T={0.2,0.4,0.8,1.6}. The model is a third order cubic polynomial on the square of the RMS value, $RMS^{2}=at^{3}$, as proposed in Ref. [29]. The goodness-of-fit statistics used to evaluate the model is the sum of squares due to error ϵ (mm, in parenthesis).

The increase in the estimation error with the simple double integral or CMS method, Figure 4a, follows the expected pattern of cubic growth with time. This growth is correlated to the sensor noise level stated in their datasheet (Table 3), the XS accelerometer being the one with slowest RMS growth, followed by the LS and lastly the WR sensor. We will denote this sensor’s classification with (XS > LS > WR)${}_{CMS}^{rms}$ (meaning better results respecting the RMS error in the distance estimation with the CMS method).

For large integration times (>2 s), the cubic model of the CMS method underestimates the RMS error for every sensor. For example, with τ = 3 s, the model-predicted error magnitude is 12.6% 22.5%, and 17.1% greater than the experimental for XS, LS, and WR sensors, respectively. However, this result is compatible with the literature, as will be discussed in the following section. The LRI method, Figure 4b, although reducing the error magnitude compared to simple CMS, has a similar qualitative response.

On the contrary, the other three methods in Figure 4 exhibit a more profound modification of the CMS-LRI response to noise, as revealed by a much better model fit and a better prediction of error for large integration times (>2 s). We will refer to the three together as the three main methods (3MM). These methods succeed in their objective of reducing the RMS error growth even for the smallest integration time of t=0.1 s. Two of them, methods OFI and MSI in Figure 4c, show a very similar quantitative response, their error being less than one-third of the CMS error for 4.1 s of integration time. The error-reduction effect of the DDI method, Figure 4d, is smaller, around half the CMS error for the same 4.1 s mark. The three methods make the Accel.LS sensor behave numerically better than the Accel.XS, a fact which is also reflected in their respective cubic error model. That is, they change the sensor ranking to (LS > XS > WR)${}_{3MM}^{rms}$.

### 4.2. The Effect of Velocity in Distance Estimation

**Figure 5:**
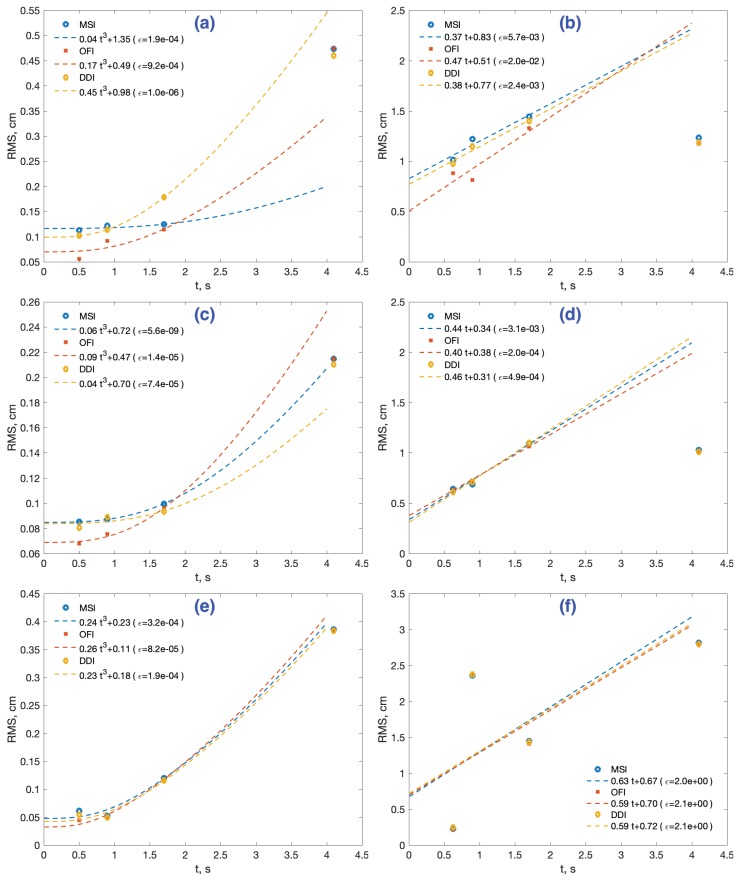
RMS position error for the Accel.XS (**first row**), Accel.LS (**second row**) and Accel.WR (**third row**) devices: numerical result of the estimations and interpolated model for small integration times (under 2 s). (**Left**) Short distance estimations. (**Right**) Large distance estimations.

The five distance estimation methods were applied with the sensors in motion at different velocities, for short distances (4 cm) and larger distances (40 cm), as prescribed in Table 2. The RMS error value was computed and the results are shown in Appendix A, Table A2, Table A3 and Table A4 for the short distance experiments, Experiments 1–4, and in Table A5, Table A6 and Table A7 for the large distance experiments, Experiments 5–8, respectively. To visualize these results, we represent the four data points corresponding to integration times of T={0.5,0.9,1.7,4.1} s, for both short and large range motions, in six diagrams in Figure 5, two for each sensor.

In the case of short range motions, the three data points T={0.5,0.9,1.7} s for the time frame of interest (up to 2 s) are used to compute a linear interpolation, using a similar third order cubic polynomial model that in the previous section, $RMS^{2}=at^{3}+b$, adding a constant term *b* that represents the significance of the error even for very small integration times. That term was not meaningful nor necessary in the static sensor experiments. As in the static case, the goodness-of-fit is evaluated with the sum of squares due to error statistic (ϵ). The last data point from the experiments (4.1 s) is used to test how the model extrapolates for large integration time data. For large range motions, a simpler linear interpolation model is proposed, RMS=at+b. We will comment on the meaning and significance of these models in the discussion section.

For the sake of clarity, we will eliminate the CMS and LRI methods from the graphics. The reason is that a mere inspection of the six tables reveals that the simple double integration CMS is clearly inferior to the other methods since it is not designed to limit the error growth. This naive approach to distance estimation produces a greater growth of error with the sensors XS < LS < WR, the same order as in the static case. It is also noticeable that the LRI method gives very bad estimations for all sensors at all integration times.

#### 4.2.1. Distance Estimation with Accel.XS

Starting with the Accel.XS sensor, the results for short range motions (4 cm) in Table A2 are represented in Figure 5a. A smaller RMS error occurs with the OFI method for the three integration times of interest (0.5, 0.9, and 1.7 s), with the greatest percentage error occurring at 1.7 s with values 3.1% (MSI), 2.85% (OFI), and 4.46% (DDI). The cubic interpolation shows a smaller constant value *b* in the OFI model, suggesting that this method could present a more robust behavior in short integration times. The model adjustment is good for the three methods, slightly better for the DDI method. For larger integration times, T>2 s, the models predict smaller errors with the MSI method and larger errors with DDI. However, the experiments at T=4.1 s indicate an outcome similar to the three methods, RMS ≈ 0.47 cm), resulting in large model prediction errors: −57% (MSI), −23% (OFI), and 26% (DDI).

The behavior of the Accel.XS sensor in large range motions (40 cm) in Table A5 is represented in Figure 5b. Again, the OFI method performs the best for short integration times. The greatest percentage error occurs at 1.7 s with similar values for the three methods: 3.61% (MSI), 3.32% (OFI), and 3.49% (DDI). The three models predict a very similar extrapolation value for large integration times (T = 4.1 s), as in fact happens in the experiments, but with an overestimation close to 90%. The RMS error at 4.1 s is even smaller than the error with only 1.7 s of integration, a counterintuitive result that will be discussed in the next section.

#### 4.2.2. Distance Estimation with Accel.LS

The Accel.LS device performs worse than the Accel.XS with the simple CMS method at low velocities (see Table A3). However, as in the case of a stationary sensor, the three main methods make it behave better when used as a distance estimator. The OFI method has better results in two of the three integration times (0.5 and 0.9 s), Figure 5c, with the greatest percentage error occurring at 1.7 s with values of 2.49% (MSI), 2.40% (OFI), and 2.34% (DDI). The cubic interpolation fitness is even better than with the Accel.XS sensor. For larger integration times, where the three methods produce very similar results in the experiments (RMS ≈ 0.21 cm), the model predictions are better than those of the Accel.XS sensor: 5% for MSI, −11% for DDI, and 23% for the OFI method.

The Accel.LS sensor in large range motions (40 cm), Table A6 or Figure 5d, produces results qualitatively similar to the XS device and are better numerically for every method and integration time. The greatest percentage error occurs at 1.7 s with values of 2.74% (MSI), 2.66% (OFI), and 2.75% (DDI). Additionally, the linear model shows a bad adjustment for a large integration time 4.1 s, with the estimation errors similar to those of the three methods (RMS ≈ 1.02 cm), but an overestimation close to 100%.

#### 4.2.3. Distance Estimation with Accel.WR

The Accel.WR device is the lowest cost device of the three tested, with a noise level and a measurement range around four times larger than the previous Accel.LS device. This is confirmed with the simple CMS method in a short distance or with low velocities, Table A4 or Figure 5f, whose RMS errors duplicate those of the previous device. The three main methods reduce RMS levels as expected, presenting almost identical results, with the greatest percentage error occurring at 1.7 s with values of 3.00% (MSI), 2.96% (OFI), and 2.89% (DDI). The cubic model shows a very good adjustment, and the results in T=4.1 s, which tend to be similar to those of the three methods (RMS ≈ 0.39 cm), present a deviation from the model prediction by 5% (MSI), 9% (OFI), and 5% (DDI).

The estimations for large range or higher speed motions, compiled in Table A7 and represented in Figure 5g, are the worst from the three sensors, for all integration times. Unexpected results occur with the smallest (*t* = 0.5 s), Experiment 8 at 100 cm/s, which showed a very low RMS value (≈0.2 cm), as well as the following at 50 cm/s, with an abnormally high value (≈2.3 cm). This behavior is independent of the 3MM method applied.

That is, the greatest error occurs at 0.9 s instead of 1.7 s , with values of 5.90% (MSI), 5.94% (OFI), and 5.94% (DDI). At 1.7 s , the error decreases to around 3.6% for all methods. Because of this inconsistency in the error behavior, the linear model fitness is even worse than that with the other two sensors. An explanation for these anomalies will be given in the following section.

## 5. Discussion

### 5.1. The Effect of Noise in Distance Estimation

The growth of distance error from an accelerometer at rest can only be explained as the effect of stochastic sources of noise from the sensor. The error caused by noise integration can be considered as a lower bound of distance estimation. This effect has been analyzed thoroughly by Thong et al. [9]. They propose a mathematical model of the growth of the RMS error with integration time *T*, which depends on three parameters: the sampling frequency $f_{s}$ (or number of samples $N=f_{s}T$), the cut frequency of the internal anti-aliasing filter of the device $f_{c}$, and the sensor noise power spectral density as given approximately by its datasheets $\sigma_{c}^{2}$. After some simplifications (see Appendix B), the model can be reduced to a cubic polynomy in the square of the RMS error of the estimated distance *d*:

(4) $$RMS{\left(d\right)}^{2}=E\left[d^{2}\right]\approx f\left(x\right)\sigma_{c}^{2}T^{3}=C_{\sigma }T^{3}$$

$x=\frac{f_{c}}{f_{s}}$ being the operation point, which depends on the specific election of cut-off and sampling frequencies, and the function f(x):

(5) $$f\left(x\right)=\frac{\pi }{24}\frac{1+e^{-2\pi x}}{1-e^{-2\pi x}}x,$$

which represents a correction factor that steps down the sensor analog noise level in the datasheets, $\sigma_{c}^{2}$, to discrete equivalent $C_{\sigma }$, which depends on the operation point. Notice that the Accel.XS sensor has a smaller value of its f(x) coefficient than the other two sensors (20% smaller).

The Thong model presented good agreement with experimental data at up to 1 s of integration, and it started to progressively underestimate the positional error with integration time, reaching a 22% error at *T* = 3.33 s [29]. We reported similar results for the CMS method in Figure 4 a, with the three sensors. The experimentally adjusted model, $RMS{\left(d\right)}^{2}=at^{3}$, shows coefficients a= 0.55, 0.94, and 3.95 for each sensor, respectively. These values correlate with their respective digital noise level $\sigma_{d}$ in Table 3, taking into account the smaller value (20%) of the Accel.XS f(x) coefficient.

The results also indicate that the three main methods show a smaller RMS error growth for any tested sensor, as expected. The DDI method is second-class from this point of view, with errors 100% greater.

As far as the model is concerned, the three main methods show an improved prediction capability. For example, for the three second distance estimation mark, the error underestimation with the CMS method was 12,6%, 22.5%, and 17.1% for the three sensors XS, LS, and WR, respectively. These numbers decrease to 6.7%, 14.7%, and 1.9% for the MSI method, 8.2%, 16.8%, and 3.1% for the OFI method, and 7.9%, 2.3%, and 2.1% for the DDI method. That capability could be useful to make model-based corrections of distance estimations in applications needing larger integration times than those addressed in this work.

Another effect found with the three error-reduction methods is to make the Accel.LS perform better than the Accel.XS sensor. What is expected from the standard deviation of the sampled data specification, $\sigma_{d}$ in Table 3, is a slower RMS growth of the latter, as is the case applying the CMS method. The reason why this tendency is reversed by those methods is not clearly understood. One explanation could be related to the parameter $\sigma_{d}$, which comes from applying a bandwidth parameter $f_{c}$ that is not clearly defined. If the real bandwidth were closer for both sensors, the lower analogue standard deviation $\sigma_{c}$ in the Accel.LS sensor could explain the results, except for the CMS method. The different sampling rate used with both sensors could be the root of this result, but this possibility was discarded with additional experiments.

### 5.2. The Effect of Motion in Distance Estimation

If for a static sensor its performance as a distance estimator correlates to the sensor quality, as given by its noise level and bandwidth, for a sensor in motion other factors are more relevant. In this work, we defined eight motions, grouped in two sets with integration times (0.5, 0.9, 1.7, and 4.1 s): short distance motion (4 cm) traversed at decreasing average speeds (10, 5, 2.5, and 1 cm/s) and long distance motion (40 cm) traversed at higher speeds (100, 50, 25, and 10 cm/s). The results reveal that distance estimation shows remarkable differences in its qualitative behavior for both groups.

#### 5.2.1. The Case of Short Distances or Low Average Speed Motion

For short distance experiments (4 cm), the CMS method generates a ranking of sensors similar to that one in the static case, (XS > LS > WR)${}_{CMS}^{rms}$. It might suggest that the XS sensor is, in principle, the best election for short-distance estimation. However, for the three main noise-reduction methods, which presented a very similar response among them, the sensor ranking reorders to (WR > LS > XS)${}_{3MM}^{rms}$ for very short integration times (0.5 and 0.9 s), and to (LS > WR > XS)${}_{3MM}^{rms}$ for times (1.7 and 4.1 s).

**Figure 6:**
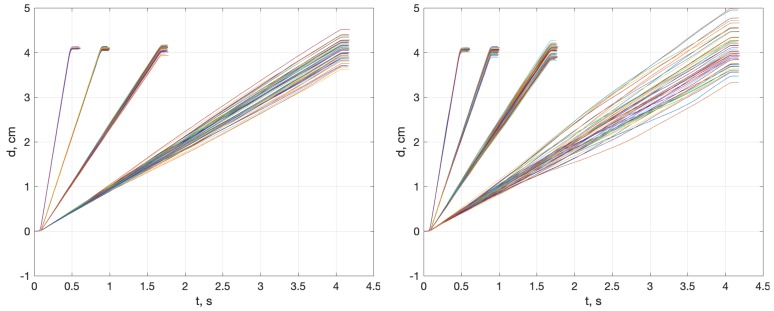
Estimated short range distance profiles for sensors Accel.LS (**left**) and Accel.WR (**right**), for the four integration times and the MSI method. For short integration times (0.5 and 0.9 s), a better mean error of Accel.WR masks its greater variance in the final RMS value. Example at 0.9 s: Accel.WR: *rms* = 0.05 cm, mean = 0.02 cm, *var* = 0.05 cm; Accel.LS: *rms* = 0.09 cm, mean = 0.09 cm, *var* = 0.02 cm.

This confusing behavior can be explained considering that the RMS value aggregates two magnitudes: the mean error and the data variance. For most combinations of methods and experiments, the Accel.WR device shows a greater experimental variance than Accel.LS, (LS > WR > XS)${}_{3MM}^{var}$. However, Accel.WR also exhibits a smaller mean error than the other two sensors in most cases (except at the 4.1 s data point). For short integration times (0.5 and 0.9 s), the variance is not large enough to degrade the better mean error, and it prevails in the final RMS value. From 1.7 s onwards, this is not the case, and both the mean and variance errors are better in the Accel.LS sensor, as is the RMS value. Figure 6 illustrates this combined effect of mean and variance. The same grounds explain the misleading RMS results with the CMS method, whose large mean errors disguise the real variance growth with time, ${\left(LS>WR≳XS\right)}_{CMS}^{var}$.

The conclusion is that the LS sensor is the best election for distance estimation. However, the behavior of the mean error is still it is left to be explained the behaviour of the mean error to be explained, which could make the WR sensor preferable over LS when mean motion velocities are faster (>2.5 cm/s) or integration times are short (<1 s). Notice that these conditions can be frequently met in biomechanics applications related to COM measurements.

**Figure 7:**
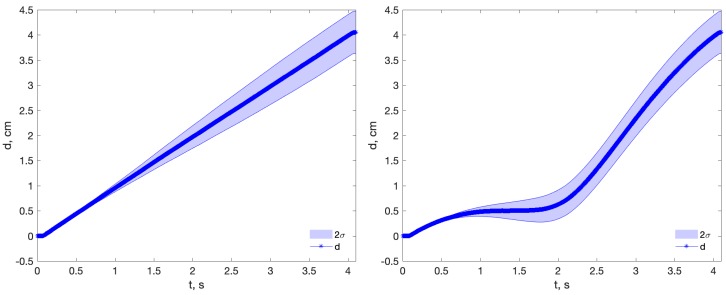
Estimated distance profile using methods MSI (**left**) and OFI (**right**) in Experiment 1 and Accel.LS sensor. Although the final RMS are similar in both methods, the distance profile of the OFI method is unrealistic.

The three main methods tend to produce similar estimations for a given sensor. The reason for that is that the DDI method tends to be similar to MSI for long integration times, because the level removal converges to a mean subtraction. The OFI method appears to be slightly better for short integration times, but its complete estimated distance profile is unrealistic, as Figure 7 reveals, calling into question the possible superiority of the method.

Another conclusion is that the cubic model of the RMS growth is only an effective predictor for a larger integration time (4.1 s) in the case of the WR sensor. It is also true with the combination LS sensor/MSI method. How to use this for model-based error reduction is not clear and needs further research.

#### 5.2.2. The Case of Long Distances or High Average Speed Motion

The results for long distance experiments (40 cm) are more troubling. To start, the simple CMS method does not allow a clear classification of the sensors from the RMS error point of view. The pattern of the variance growth is the same as that in short range, ${\left(LS>WR>XS\right)}_{all}^{var}$. Thus, the RMS differences with the CMS method must come from a more dispersed behavior of the mean error.

The three main methods, which again presented a similar response among them, rank the sensors differently: for 0.5 s integration times the order is (WR > LS > XS)${}_{3MM}^{rms}$, and for times (0.9, 1.7, and 4.1 s) it is (LS > XS > WR)${}_{3MM}^{rms}$. That is, for all cases under 50 cm/s, the LS outperforms the WR sensor as before. Even the XS sensor performs better, because WR is no longer the sensor with the smallest mean error, and the mean value produces (XS > LS > WR)${}_{3MM}^{avg}$.

**Figure 8:**
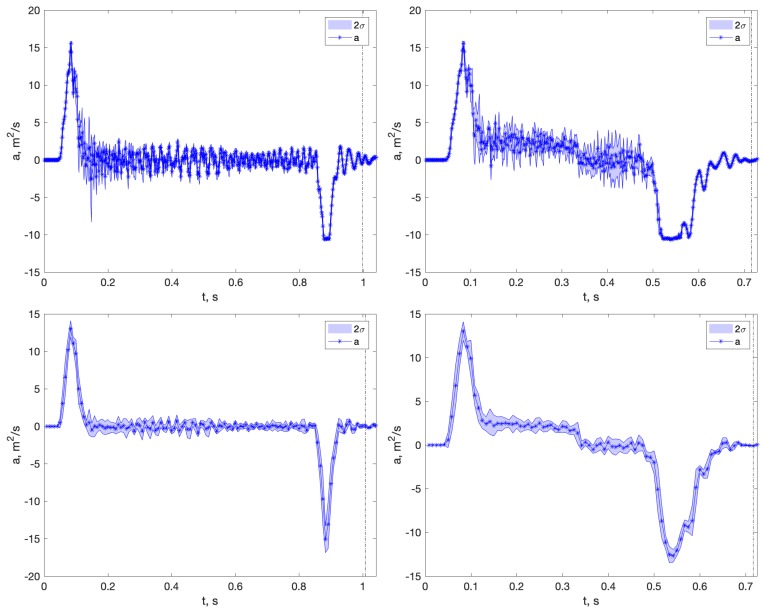
Average accelerometry profile of Accel.WR (**first row**) and Accel.XS (**second row**), with a band corresponding to two standard deviations. (**Left**) Experiment 7 at 50 cm/s. (**Right**) Experiment 8 at 100 cm/s.

As a consequence, the possibility that the WR could be preferable is restricted only to Experiment 8 at 100 cm/s. The WR sensor has no saturation problems, but its deceleration peak seems truncated around −10 m/s${}^{2}$ in both experiments, see Figure 8. The reason for this is not clear, and further research is required. The XS sensor in Experiment 7 shows a large deceleration peak, which could correspond to the 30 Hz filtered real deceleration. In Experiment 8, the peaks are lower, but that can be explained by the fact that, at 100 cm/s, the robot works within its acceleration limits, and the change in velocity takes 0.3 s instead of 0.1 s, greatly reducing the effect of the sensor-limited bandwidth.

Hence, the three sensors registered different motions in the last two experiments (Experiments 7 and 8). A measure of this difference is the mean value of the accelerometer peaks in the experiments, Table 5. For Experiments 1 to 6, the mean peak is similar for LS and WR sensors, and it is downscaled for the XS sensor. This is so because the Accel.XS has a bandwidth of 30 Hz, and the robot’s change in velocity occurs at ≈0.1 s, implying that acceleration has 10 Hz components that are attenuated by its filter. This can explain why the Accel.XS, which has a good performance in static, ranks last in motion-related experiments.

However, in Experiments 7 and 8 the mean deceleration peak is very different for the three devices.

The actual motion profile carried out by the robot was the same for all sensors, so they failed to register the actual acceleration. Under those circumstances, rejecting those two experiments from the point of view of a fair evaluation of methods is unavoidable. The conclusion to be drawn is that the two first data points in Figure 5b,d,f are invalid, so we do not have enough data to build a model to predict errors for larger (>2 s) integration times.

To explain why the model fails in these cases, it is necessary to inspect individual raw signals with detail. The actual robot accelerations in Cartesian space are not easy to obtain, but a first inspection suggests that they can rise over the 2 g limit during the acceleration peak in Experiments 7 and 8, saturating the LS sensor. It is not clear how an MEMS accelerometer reacts to saturation, but in any case the distance estimation results obtained are subject to objections.

For a given sensor, any of the main methods produce similar results, and the same reasoning made in short distances is valid for the preference of DDI or MSI over OFI. Another outcome of this study is that, for integration times under 2 s, the worst-case RMS error stays in a range between 2 and 4% in percentage, always with a longer time 1.7 s. This is true for all main methods and sensors. Therefore, the average speed of the motion is not, in itself, a key factor for distance estimation.

## 6. Conclusions

The measurement of distances using accelerometers is a functionality that is used in many HMS applications. The context of these measurements is an integration time of less than 2 s, and a movement length of up to 2 m. Even in these restricted conditions, it is advisable that the estimation algorithm incorporates some mechanism to limit the growth of the error, which, being quadratic in nature, deteriorates the estimates even for such small integration times.

In this work, we have compared five methods with three different sensors in controlled experimental conditions, which reflect those found in practice in HMS. We have separated these conditions into two groups: short and long distances. The results obtained are as follows: (1) With the static sensor, the RMS error is related to the quality of the sensor in terms of bandwidth, noise density, and sampling frequency. The algorithms to reduce the error can reduce it by up to one-third. The simple elimination of the mean (MSI method) produces optimal results. The XS and LS sensors have a similar response, better than the WR. (2) With the sensor in motion, the expected error (RMS) in distance estimation is below 4.5% in 1.7 s for both distance ranges, whatever the sensor used. (3) The tested methods produce quite similar results in both groups of experiments. The OFI is sometimes better with low periods of integration, but no general rule can be defined in this respect. (4) The best sensor to estimate distances turns out to be the LS at all distances. For long distances and average velocities over 50 cm/s, the measurements are no longer reliable in all three devices.

The study confirms that it is feasible to use accelerometers to estimate short linear displacements of the body. More than the estimation method applied, the motion kinematic conditions can be a key factor in the performance of this estimation, combined with the type of accelerometer used, and those factors have to be jointly evaluated.

## Figures and Tables

**Table 1:** Kinematic restrictions of the spatial gait parameters (displacements) of interest.

Human Motion Displacement	Range	Time Range	Average Speed
stride length	94–185 cm	1.6–0.8 s	60–230 cm/s
displacement of vCOM	2.2–6 cm	0.5–0.9 s	4.4–12 cm/s
displacement of mlCOM	8–2.4 cm	0.5–0.9 s	2.6–16 cm/s

**Table 2:** Range of motion conditions for the eight designed experiments: four integration times to measure short and range distances, with increasing average speeds.

ID	Distance *D* [cm]	Speed *v* [cm/s]	Integration Time τ=v/D [s]
0	0	0	0.4–4
1	4	1	4
2	4	2.5	1.6
3	4	5	0.8
4	4	10	0.4
5	40	10	4
6	40	25	1.6
7	40	50	0.8
8	40	100	0.4

**Table 3:** Accelerometer characteristics from their datasheet.

ID	Model	Analog Noise	BW	Dig. Noise	Range	Sampling
		$\sigma_{c}$ [$\mu g/\sqrt{\mathrm{Hz}}$]	$f_{c}$ [Hz]	$\sigma_{d}$ [${\mathrm{cm}/s}^{2}$]	[g]	$f_{s}$ [Hz]
Accel.XS	MTi ADXL206	110	30	0.74	±5	120
Accel.LS	KXRB5-2042	45	200	0.78	±2	512
Accel.WR	LSM303DLHC	220	200	3.82	±8	512

**Table 4:** Methods for distance estimation and correction.

Method ID	Integration Method	Reference	Distance Range [cm]
CMS	Cumulative	Bamberg [14]	stride length (foot)
LRI	Lineal Reset	Sabatini [15]	stride length (foot)
MSI	Mean subtraction	Alvarez [8,16]	stride length (foot, vCOM)
OFI	Optimally filtered	Kose [17]	stride length (apCOM)
DDI	De-drifted	Rampp [19]	stride length (foot)

**Table 5:** Acceleration and deceleration average peaks (m/s${}^{2}$) for every experiment and accelerometer.

Acc.peak	Exp. 1	Exp. 2	Exp. 3	Exp. 4	Exp. 5	Exp. 6	Exp. 7	Exp. 8
xs${}_{max}$	0.5835	1.1472	1.7933	3.0520	3.6420	7.9899	13.0096	13.0484
xs${}_{min}$	−0.3011	−0.7410	−1.3885	−2.4647	−2.1949	−6.5669	−15.4089	−12.9085
ls${}_{max}$	0.9805	1.6469	2.2670	3.3490	4.0066	9.1446	15.6015	15.5820
ls${}_{min}$	−0.4294	−0.9382	−1.5896	−2.7457	−2.4712	−7.9310	−13.6501	−12.8897
wr${}_{max}$	0.9986	1.6834	2.3463	3.2971	4.0536	9.1247	15.5784	15.5756
wr${}_{min}$	−0.4785	−0.8659	−1.5780	−2.7507	−2.4107	−7.9810	−10.6316	−10.6652

## Appendix A. Numerical Results of the Experiments

**Table A1:** RMS position error growth (in mm) with integration time.

Integration Time	Sensor	CMS	LRI	MSI	OFI	DDI
	Accel.XS	0.0191	0.0058	0.0083	0.0085	0.0082
0.1 s	Accel.LN	0.0148	0.0050	0.0062	0.0062	0.0122
	Accel.WR	0.0477	0.0163	0.0218	0.0220	0.0249
	Accel.XS	0.0528	0.0177	0.0226	0.0228	0.0338
0.2 s	Accel.LN	0.0457	0.0159	0.0173	0.0178	0.0353
	Accel.WR	0.1377	0.0500	0.0643	0.0652	0.0948
	Accel.XS	0.1534	0.0543	0.0642	0.0650	0.1187
0.4 s	Accel.LN	0.1509	0.0522	0.0478	0.0498	0.1042
	Accel.WR	0.4011	0.1487	0.1841	0.1874	0.3367
	Accel.XS	0.4656	0.1674	0.1848	0.1902	0.3770
0.8 s	Accel.LN	0.5338	0.1828	0.1442	0.1541	0.2929
	Accel.WR	1.2358	0.4521	0.5158	0.5285	1.0293
	Accel.XS	1.5094	0.5402	0.5071	0.5259	1.1020
1.6 s	Accel.LN	1.9735	0.6718	0.4521	0.4964	0.8792
	Accel.WR	4.0304	1.4490	1.4543	1.5118	3.3102
	Accel.XS	2.2098	0.7924	0.7122	0.7417	1.5532
2.0 s	Accel.LN	3.0168	1.0275	0.6698	0.7410	1.2942
	Accel.WR	5.9978	2.1388	2.0653	2.1575	4.5433
	Accel.XS	4.4258	1.5908	1.3955	1.4726	3.0711
3.0 s	Accel.LN	6.5185	2.2193	1.3588	1.5290	2.3091
	Accel.WR	12.4515	4.3786	3.8081	4.0032	8.6623
	Accel.XS	7.3514	2.6230	2.1621	2.2899	4.5301
4.0 s	Accel.LN	11.2698	3.8381	2.2422	2.5659	3.5671
	Accel.WR	21.2614	7.3826	5.9158	6.3202	12.9859

Results of the experiments in Table 2: RMS position error growth (cm) with integration time (s) for the five estimation methods.

**Table A2:** Accel.XS in short distance.

	CMS	LRI	MSI	OFI	DDI
0.5	0.10	1.89	0.11	0.06	0.10
0.9	0.26	2.05	0.12	0.09	0.11
1.7	1.10	2.37	0.12	0.11	0.18
4.1	6.51	4.17	0.47	0.47	0.46

**Table A3:** Accel.LN in short distance.

	CMS	LRI	MSI	OFI	DDI
0.5	0.08	1.91	0.09	0.07	0.08
0.9	0.43	2.13	0.09	0.08	0.09
1.7	1.88	2.66	0.10	0.10	0.09
4.1	9.93	5.39	0.21	0.21	0.21

**Table A4:** Accel.WR in short distance.

	CMS	LRI	MSI	OFI	DDI
0.5	0.13	1.92	0.06	0.04	0.05
0.9	0.73	2.24	0.05	0.05	0.05
1.7	3.07	3.07	0.12	0.12	0.12
4.1	20.26	8.96	0.39	0.39	0.38

**Table A5:** Accel.XS in large distance.

	CMS	LRI	MSI	OFI	DDI
0.5	1.04	18.40	1.01	0.88	0.98
0.9	2.25	18.33	1.22	0.82	1.15
1.7	2.70	19.04	1.44	1.33	1.40
4.1	5.43	21.28	1.23	1.17	1.19

**Table A6:** Accel.LN in large distance.

	CMS	LRI	MSI	OFI	DDI
0.5	1.15	18.41	0.64	0.64	0.61
0.9	0.80	18.49	0.69	0.73	0.71
1.7	0.38	19.12	1.09	1.06	1.10
4.1	8.94	22.46	1.03	1.02	1.01

**Table A7:** Accel.WR in large distance.

	CMS	LRI	MSI	OFI	DDI
0.5	1.69	18.32	0.23	0.21	0.25
0.9	1.23	18.40	2.36	2.38	2.38
1.7	1.69	18.47	1.45	1.41	1.44
4.1	6.99	21.20	2.82	2.81	2.80

## Appendix B. Model of the Accelerometer Noise Effect in Position Estimation

For a static sensor at rest, *N* samples are collected with a sampling frequency $f_{s}$ Hz, and the RMS error of the final position estimation S(N) is calculated by the square root of its expectation value of the mean, $RMS\left[S\left[N\right]\right]=\sqrt{E[{\left(S\left[N\right]\right)}^{2}]}$. With this setup, the RMS error is given by ([29], Equation (55))

$$\begin{array}{c} E[S{\left(N\right)}^{2}]=\\ \;\frac{r[0]}{12f_{s}^{4}}\left(N-1\right)\left(N^{2}-2N+3\right)+\frac{A}{3f_{s}^{4}}S_{3}\left(N-2\right)-\\ \;\frac{A}{6f_{s}^{4}}\left(3N^{2}-6N+5\right)S_{1}\left(N-2\right)+\\ \;\frac{A}{6f_{s}^{4}}\left(N-1\right)\left(N^{2}-2N+3\right)S_{0}\left(N-2\right) \end{array}$$

where r[k] is the *k*th lag of the autocorrelation function, which can be written as

(A1) $$r\left[k\right]=Ae^{\left(-\beta k\right)},\;A=\frac{\pi f_{c}\sigma^{2}}{2},\;\beta =\frac{2\pi f_{c}}{f_{s}}$$

the terms $S_{i}\left(N-2\right)$ being the sums of decaying exponentials depending on the number of samples *N*:

$$\begin{array}{ccc} S_{0}\left(N\right) & = & \sum_{n=1}^{N}exp\left(-\beta n\right)\\ S_{1}\left(N\right) & = & \sum_{n=1}^{N}n\cdot exp\left(-\beta n\right)\\ S_{3}\left(N\right) & = & \sum_{n=1}^{N}n^{3}\cdot exp\left(-\beta n\right). \end{array}$$

This expression of the RMS error in position estimation is not easy to evaluate. Notice that it depends not only on the integration time but also on the ratio $f_{s}/f_{c}$. They can be simplified for further analysis.

Firstly, the previous summations of exponentials can be simplified assuming that N>>1, and they can be reduced to

(A2) $$\begin{array}{ccc} S_{1}\left(N\right) & = & -\frac{\partial S_{0}\left(N\right)}{\partial \beta } \end{array}$$

(A3) $$\begin{array}{ccc} S_{3}\left(N\right) & = & -\frac{\partial^{3}S_{0}\left(N\right)}{\partial \beta^{3}}. \end{array}$$

$S_{0}\left(N\right)$ can be directly evaluated as

(A4) $$S_{0}\left(N\right)=\frac{exp(-\beta )\cdot (1-exp(-\beta N))}{1-exp(-\beta )}$$

leading to the formula

(A5) $$S_{1}\left(N\right)=-\frac{exp(-\beta )-exp(-\beta (N+1))-Nexp(-\beta (N+1)+Nexp(-\beta (N+2)))}{{\left(exp\left(-\beta \right)-1\right)}^{2}}.$$

The terms exp(−βN) with a large enough *N* will be negligible, as opposed to exp(−β). Bearing this in mind, the approximate sums will remain in the following form:

(A6) $$\begin{array}{ccc} S_{0} & \approx & \frac{exp(-\beta )}{1-exp(-\beta )}\\ S_{1} & \approx & \frac{exp(-\beta )}{{\left(exp\left(-\beta \right)-1\right)}^{2}}\\ S_{3} & \approx & \frac{exp(-\beta )+4exp(-2\beta )+exp(-3\beta )}{{\left(exp\left(-\beta \right)-1\right)}^{4}}. \end{array}$$

Placing these terms in Equation (A1), it can be re-arranged by naming $B\left(N\right)=\left(N-1\right)\left(N^{2}-2N+3\right)$, and grouping in terms, becoming

(A7) $$E\left[S{\left(N\right)}^{2}\right]=\frac{A}{3f_{s}^{4}}\left[\frac{B\left(N\right)+2B\left(N\right)S_{0}}{4}+S_{3}-\frac{3N^{2}-6N+5}{2}S_{1}\right]$$

making clear that the constant term $S_{3}$ can be neglected against the other terms which grow with *N*, and the expected value of the square error in position becomes

(A8) $$E\left[S{\left(N\right)}^{2}\right]=\frac{A}{6f_{s}^{4}}\left[\frac{B\left(N\right)+2B\left(N\right)S_{0}}{2}-\left(3N^{2}-6N+5\right)S_{1}\right].$$

Including B(N) and re-grouping terms leads to

(A9) $$\begin{array}{ccc} E[S{\left(N\right)}^{2}] & = & \frac{A}{6f_{s}^{4}}\left[\left[\frac{1+2S_{0}}{2}\right]N^{3}-\left[\frac{3}{2}\left(1+2S_{0}\right)+3S_{1}\right]N^{2}+\right.\\ & & \left.+\left[\frac{5}{2}\left(1+2S_{0}\right)+6S_{1}\right]N-\left[\frac{3}{2}\left(1+2S_{0}\right)+5S_{1}\right]\right]. \end{array}$$

Again, factorial analysis of this expression reveals that the terms in $N^{2}$, the term in *N*, and the independent term can be neglected, leading finally to

(A10) $$E\left[S{\left(N\right)}^{2}\right]\approx \frac{A}{12f_{s}^{4}}\left(1+2S_{0}\right)N^{3}=f\left(x\right)\sigma_{c}^{2}T^{3}$$

where

(A11) $$\begin{array}{ccc} x & = & \frac{f_{c}}{f_{s}} \end{array}$$

(A12) $$\begin{array}{ccc} f(x) & = & \frac{\pi }{24}\frac{1+exp(-2\pi x)}{1-exp(-2\pi x)}x. \end{array}$$

The function f(x) can be understood as a correction factor depending on the operation point, which steps down the sensor analog noise level in the datasheets $\sigma_{c}^{2}$ to a nominal discrete equivalent $C_{\sigma }$. This factor approaches the linear relation $f\left(x\right)=\frac{\pi }{24}x$ when $f_{c}\geq 2f_{s}$.
